# Foehn wind as a biometeorological stressor: effects on somatic and mental health – a narrative review

**DOI:** 10.1007/s00484-026-03274-6

**Published:** 2026-07-16

**Authors:** Zbigniew Pankiewicz, Szymon Florek, Robert Pudlo

**Affiliations:** Department of Psychoprophilaxis, Faculty of Medical Sciences in Zabrze, Medical University of Silesia in Katowice, Katowice, Poland

**Keywords:** Foehn wind, Biometeorology, Weather-related stress, Mental well-being, Time-lag effects, Climate change

## Abstract

This review summarizes research on foehn wind as a complex biometeorological stressor affecting somatic and neuropsychological health with particular attention to time-lagged effects and vulnerable populations. Foehn, warm and dry winds descending mountains, are linked to decreased mental well-being and are colloquially termed “suicide winds” in regional folklore; however, such interpretations require scientific verification. A structured literature search and narrative review of PubMed, Web of Science, Scopus, Researchgate and Google Scholar databases, using “foehn”, “halny” and “health” keywords in Polish and English, was conducted. Scientific articles, clinical reports, and reviews were analyzed. Studies, though limited in number, feature long observation periods and numerous participants. Research indicates a correlation between halny occurrences and increased suicide rates, aggression, and mental health deterioration, especially in predisposed individuals. Halny appears to exacerbate psychopathology during mental health crises, with stronger effects correlating with more pronounced winds. Effects are observed pre- and post-halny. While studies show a correlation between halny and worsened mental health, a direct causal link is not definitively established. Further detailed research is necessary to confirm causality and understand the mechanisms involved. This research would facilitate effective preventive measures, crucial given potential increases in halny frequency due to climate change.

## Introduction

The belief in the impact of weather changes on overall human well-being has been deeply rooted in society for centuries. Hippocrates, in his work “On Airs, Waters, and Places,” wrote about the influence of meteorological conditions on health, considering the study of seasons and winds to be a crucial component of medical education (Hipokrates 1890). Currently, there is a widespread and deeply ingrained belief in the impact of atmospheric changes on mental well-being. The number of people who declare that weather has a significant impact on their well-being reaches nearly 30% of the world population (Mirković and Savić 2022), and weather changes along with climate changes are becoming increasingly violent (Hov et al. 2013), which may exacerbate the symptoms of weather-related stress and meteoropathy.

Particular attention is drawn to the phenomenon of the foehn wind (Latin: favonius, “hot south wind”), a characteristic example of a biometeorological stressor, which is common in Poland, especially in the Podhale region in the form of the halny wind. It is known by various names in other parts of the world, such as Diablo Winds (Northern California), Puelche (Chile), Zonda/Witches Wind (Argentina), Bejdewind (South Africa), Chinook (Canada, United States), etc.

Foehn wind develops under synoptic conditions that produce air advection from a direction forcing the air mass to cross an orographic barrier. In the Tatra Mountains, southerly airflow is lifted on the windward side and subsequently descends on the leeward side of the range. As the descending air warms adiabatically and becomes relatively dry, it produces the characteristic warm, dry and gusty foehn wind, accompanied by abrupt changes in temperature, humidity, wind speed and atmospheric pressure Grajek and Bednorz (2025) which together constitute a complex environmental stressor for the human organism.

The power of this phenomenon has been reflected in the works of many artists, such as in Stanisław Witkiewicz’s 1895 painting “Halny Wind” (Witkiewicz 1895), and a highland song warns “Fear will grip your hands today.” In the nomenclature of folk beliefs, the Polish foehn is often referred to as the “suicide wind,” (Bartmiński 2012) however, these cultural interpretations require verification within a scientific biometeorological framework. Analyzing the press, one can come across articles such as, “Why does the halny wind drive people crazy,” “Halny: madness and death flow from the mountains,” “Does strong wind deepen depression and lead to suicides?” “So that the halny wind takes no one,” or “The Devil’s Lullaby. Is the halny wind a wind of madness?” (Szymaszek n.d; Zając n.d; Bednarek 2021; Cieślińska n.d).

Anthropological work from the Swiss Alps shows that Foehn has long been embedded in local explanatory models of bodily and mental discomfort. In Leukerbad, residents commonly associated Foehn with headaches, depressed mood, suicidal behavior, nervousness, and a general feeling of ill health, illustrating how this wind functions not only as a meteorological phenomenon but also as a culturally recognized biometeorological stressor (Strauss 2007). Hence, it seems justified to conduct a scientific examination of the association between foehn wind exposure and human health outcomes, rather than relying on anecdotal or cultural interpretations.

The objective of this review is to provide a comprehensive summary of the current research regarding the impact of foehn wind as a biometeorological stressor on human health, including both somatic and mental health domains, with particular attention to time-lagged effects and vulnerable populations.

## Methods

We performed a narrative review supported by a structured literature search across the PubMed and Google Scholar, Web of Science, Scopus, Researchgate databases, utilizing the keywords “foehn,” “halny,” and “foehn AND health” in both English and Polish to identify relevant scientific articles, clinical reports, and reviews. Due to the limited number of available manuscripts addressing this specific topic, no restrictions were imposed on publication date.

The inclusion criteria encompassed studies directly analyzing the relationship between exposure to foehn winds and human health (both mental and somatic), presenting original data, case reports, or evidence syntheses, and available in English or Polish. Conversely, studies focusing solely on meteorological aspects, concerning other weather phenomena, lacking scientific data, or not accessible in full text were excluded.

The limited number of studies meeting the inclusion criteria was often characterized by long observation periods and numerous participants; however, methodological heterogeneity across studies represented a potential limitation. Given this heterogeneity, a narrative synthesis approach was applied rather than a formal systematic review. To further ensure comprehensive coverage, the bibliographies of identified articles and selected journals within the fields of biometeorology and environmental health were also examined.

An overview of the key studies included in this narrative review, including geographical region, type of foehn wind, health outcomes, and timing of effects, is presented in Table 1.

**Table 1:** Note: This data is mandatory, please provide

Study	Region	Health outcome	Timing of effect (lag)	Population
Przybyła 1994	Zakopane, Poland	Psychiatric emergency calls	During onset	General population
Corbett et al., 1996	Southern California, USA	Asthma exacerbations, ED visits	During onset	Emergency department patients
Maciejczak et al. 2020	Poland	Cardiac interventions	1–2 days before onset	General population
Cooke et al. 2000	Alberta, Canada	Migraine occurrence	1 day before onset	Adults with migraine
Piorecky et al. 1997	Alberta, Canada	Migraine probability	Pre-onset period	Adults, higher risk in older age
Reinsberger / Koszewska et al. 2016–2019	Austria / Poland	Suicide occurrence	0–2 days around onset	Adult population
Trepińska et al. 2005	Kraków, Poland	Suicides by hanging	During foehn influence	Urban population
Lickiewicz et al. 2020	Kraków, Poland	Aggressive behavior	−1 to + 2 days	Psychiatric inpatients
Mikutta et al. 2022	Switzerland	Mental distress (BSI dimensions)	~ 2 days after onset	Psychiatric inpatients (early stay)
Bundo et al. 2020	Bern, Switzerland	Psychiatric hospitalizations	+ 3 days	General population
Fletcher 1988	Alberta, Canada	Subjective well-being changes	During exposure	General population
Hanselmann et al., preprint 2025	Liechtenstein / Rhein valley	HR, HRV, perfusion index, sleep, respiratory rate	During exposure	714 healthy adults
Schneidewind et al. 2025	Switzerland	Emergency hospitalizations; all-cause and cause-specific	During exposure	General population

## The impact of foehn wind on somatic health

The influence of foehn wind on health is complex and not fully understood. Research findings are inconsistent, and the mechanisms behind some observed phenomena are not entirely clear. Several studies indicate that health effects may occur not only during the wind itself, but also in the period preceding or following its onset, suggesting delayed physiological responses.

Recent wearable-based observational data suggest that Foehn exposure may be associated with subtle autonomic and peripheral physiological changes. In a preprint study from Liechtenstein including 714 healthy adults monitored over 11 months using medical sensory bracelets, Foehn episodes were associated with a small increase in nightly heart rate (+ 0.25 bpm; 95% CI: 0.17 to 0.33), a decrease in heart rate variability measured as SDNN (− 0.29%; 95% CI: −0.55 to − 0.04), and changes in perfusion index (+ 0.78%; 95% CI: 0.22 to 1.33), while respiratory rate, wrist skin temperature, and sleep duration remained unchanged. These findings may support the hypothesis that Foehn acts as a mild physiological stresor. No sex-related differences in physiological responses were detected. (Hanselmann 2025)

In addition to wind speed, pressure changes, and humidity, researchers at Loma Linda University Hospital in California also noted a decrease in air pollution, which should lead to fewer asthma interventions. However, over a 4-year period from 1990 to 1993, the number of emergency department visits during Santa Ana winds was slightly higher compared to interventions during other weather conditions (3.12 vs. 2.16 visits per day, *P* < 0.0001). Admission rates were also higher, suggesting a potentially more severe course of events (21.9 vs. 18.7%, *P* < 0.05). It was found that admissions increased with the onset of Santa Ana, and decreased in the following days. However, the study did not identify a specific factor causing the increased number of asthma exacerbations (Corbett 1996).

Studies on cardiovascular events and foehn wind contradict the association of foehn wind with an increased number of cases (Maciejczak et al. 2020; Ambach et al. 1992; Gnecchi Ruscone et al. 1985). A study conducted in Poland found an increase in cardiological interventions in the days immediately preceding the occurrence of foehn wind. Interestingly, no correlation was found between other environmental variables and the number of events during this period (Maciejczak et al. 2020).

In a large Swiss case-time series study covering 1998–2019, Foehn wind intensity was not independently associated with emergency hospitalizations after adjustment for temperature. However, Foehn days appeared to amplify heat-related hospitalization risk, particularly among females, older adults, and for respiratory and mental-health-related admissions, suggesting that Foehn may act as an effect modifier of heat-related morbidity rather than as an isolated health risk factor. (Schneidewind 2025)

The conditions preceding foehn wind have been shown to have an effect on the occurrence of migraines. A study from the Canadian province of Alberta involving 75 patients found that the likelihood of migraine increased before the onset of chinook wind (odds ratio 1.24; 95% CI 1.08 to 1.42). Chinook days were also characterized by an increased risk of pain (1.19; 1.02 to 1.39). This finding was corroborated by another study on the impact of chinook winds on migraines, which found that older people were more susceptible than the rest of the population (Cooke et al. 2000; Piorecky et al. 1997).

A comprehensive study from New Zealand spanning 1968–1997 analyzed the impact of wind on sudden infant death syndrome (SIDS) but found no association between SIDS and pre-foehn or foehn days (Macey et al. 2000).

An interesting case report describes a 56-year-old man who experienced massive decompression retinopathy due to acute angle-closure glaucoma. After thorough examination, the halny wind was suggested as a possible cause, potentially leading to a decrease in intraocular pressure that may have triggered the condition (Sabal et al. 2023).

## The impact of foehn wind on mental health

There are relatively few studies on the impact of foehn wind on mental health, and it is often included as an additional factor in research. However, those that have been conducted are typically characterized by a long observation period and a large sample sizes. Foehn wind is most typically associated with behavioral and psychological stress responses, including aggression and increased suicide risk.

This belief is somewhat confirmed by a 16-year study correlating suicidal behavior with foehn wind (Koszewska et al. 2019). Foehn wind was defined as a weather phenomenon based on criteria of temperature change, wind direction and speed, cloud type and relative humidity. The period of influence was defined as the two days surrounding its occurrence.

The analysis also included days with the occurrence of individual factors, divided into subgroups of different foehn wind quality. An increased risk of suicide was noted only on days that met all the criteria and only in the summer and autumn (Koszewska et al. 2016).

One of the earliest Polish investigations addressing the relationship between weather conditions and mental health was conducted by Przybyła in Zakopane. Using records of emergency psychiatric service calls for neuroses, psychoses, schizophrenia, and suicide attempts between 1983 and 1987 together with meteorological observations, the author evaluated the influence of various weather situations, including foehn episodes, on psychiatric emergencies. The association between foehn occurrence and psychiatric emergency calls was established, although no single season showed a particularly pronounced effect (Przybyła 1994).

The impact of foehn wind on suicide risk can extend far beyond the mountainous region. Also in Kraków, about 80 km from the Tatra Mountains foothills, the number of suicides by hanging increased during the influence of foehn winds, which was studied in the years 1991–2002 (Trepińska et al. 2005). It was speculated that the increase in the number of suicides could be related to severe psychological stress caused by sudden weather changes (Trepińska et al. 2005) or higher temperatures and electromagnetic pulses generated by lightning. Additionally, the effect could be intensified by the influx of positive ions, which would result in an increased number of suicides as early as 2 days before the arrival of foehn wind, which the study did not confirm (Koszewska et al. 2019).

Another study documented a link between foehn wind and increased aggressive behavior among patients in a Kraków psychiatric hospital (Lickiewicz et al. 2020). From January 2015 to March 2017, meteorological conditions were recorded, and those that occurred on days with a greater than average number of aggressive behaviors leading to the use of physical coercion were sought.

Data analysis allowed the conclusion that the increase in interventions, among other weather conditions, was accompanied by foehn wind (especially lasting several days). Behaviors related to aggression, which led to the reaction of medical personnel, began to increase the day before the start of the weather change, and the intensity ended up two days after the end of the foehn wind. The impact on mental state during hospitalization was also found in patients hospitalized in Switzerland (Mikutta et al. 2022).

During Föhn winds, a brief symptom inventory (BSI) was conducted, which revealed deterioration in dimensions such as paranoid thinking, interpersonal relationships, obsessive-compulsive symptoms, depression, anxiety, and phobic anxiety among patients beginning their stay in the psychiatric ward.

All health effects showed a two-day delay to the foehn wind and, interestingly, only concerned patients in the early stages of hospitalization.

Patients ending their stay showed resistance to weather influences. This difference led to the conclusion that meteorological stressors, such as foehn wind, have an effect on people with increased psychological sensitivity, but not on people who have stabilized during hospitalization.

Weather conditions that worsen the mental state should also lead to increased hospital admissions. This thesis was reflected in a study by M. Bundo, in which data collected over a period of 35 years, including 71,931 cases of hospitalization, showed a link between foehn wind and an increase in the number of hospitalizations (Bundo et al. 2020). Admissions with a 3-day delay in relation to the studied atmospheric phenomenon were considered to be the result of the wind’s influence.

More recent Swiss evidence adds nuance to these findings: although Foehn winds were not independently associated with mental-health-related emergency hospitalizations, mental-health admissions showed one of the strongest increases in heat-related hospitalization risk on Foehn days compared with non-Foehn days (Schneidewind 2025).

The widely attributed impact of foehn wind on well-being was also studied among residents of southern Alberta (Fletcher 1988). A survey of 1,828 residents found a discrepancy between the reactions of women and men.

As anticipated, there was an increase in irritability among males during prolonged exposure to foehn wind. The results for women were less obvious. The only observed dependency was a lower than average intensity of fatigue, headache, low mood, and irritability shortly before the arrival of Chinook. The authors attributed this discrepancy between genders to variations in hormonal economy. It is noteworthy that a study conducted in Switzerland found no disparities in the impact on patients of different genders (Bundo et al. 2020). Whereas in a study conducted by M. Bundo, the impact of foehn wind on women was slightly greater, but only in the age group from 65 years of age upwards.

The Canadian Chinook had a weaker impact on residents of the region with a longer history of living in the study region. People who declared themselves to be weather-sensitive did not feel the effects of weather changes more often than people who did not report such sensitivity (Fletcher 1988).

## Conclusions

Foehn wind can have a negative impact on mental health. Studies have shown a correlation between the occurrence of foehn wind and an increase in the number of suicides, aggressive behaviors, or worsening of mental state among various study populations, mainly among people previously predisposed to the above. For individuals experiencing a mental health crisis, the foehn wind appears to exacerbate the course of psychopathology, which in the case of people struggling with suicidal thoughts, may lead to a tragic outcome. In certain studies, it has been demonstrated that women possess heightened sensitivities, and exposure to wind may have a beneficial effect on them, reducing fatigue, headache, or irritability.

The mechanism by which foehn winds affect mental health is not fully understood. The proposed pathophysiological mechanisms encompass a synergy of rapid adiabatic transformations, putative effects of air ionization on neurobehavioral regulation, and autonomic nervous system (ANS) destabilization induced by sferics and barometric micro-oscillations (Krueger and Reed 1976; Sulman 1980). However, it has been demonstrated that the more fully articulated this phenomenon, the greater its impact.

Recent epidemiological evidence also indicates that Foehn-related health effects may be context-dependent and may become more apparent when Foehn coincides with other environmental stressors, particularly heat. This supports a model in which Foehn is not necessarily an independent causal factor, but may act as an amplifier of existing physiological and psychiatric vulnerability under adverse thermal conditions.

The area where the influence of foehn wind can be expected, as well as the body’s reaction time to this change, also seems to be difficult to determine. It is known that the effects are being studied from a few days before the foehn wind, through its duration to a few days after its end.

Although a link has been established between the occurrence of foehn winds and a worsening of mental health, it cannot be said with certainty that foehn winds are the cause of these problems. Therefore, further research is needed, including a more detailed analysis of the phenomena associated with this biometeorological event, in order to confirm the causal relationship and establish the mechanisms by which this infamous wind, affects mental health.

The potential benefit of such work is the possibility of implementing appropriate prevention for a predictable environmental stressor that affects the well-being of many people, which may become an increasingly common problem with ongoing climate change.

## Data Availability

No new data were generated or analysed in this study. Data sharing is not applicable to this article.
